# Targeting Signaling Pathway Networks in Several Malignant Tumors: Progresses and Challenges

**DOI:** 10.3389/fphar.2021.675675

**Published:** 2021-05-31

**Authors:** Hongdan He, Xiaoni Shao, Yanan Li, Ribu Gihu, Haochen Xie, Junfu Zhou, Hengxiu Yan

**Affiliations:** ^1^ Qinghai Tibet Plateau Research Institute, Southwest Minzu University, Chengdu, China; ^2^ Immunotherapy Laboratory, College of Pharmacology, Southwest Minzu University, Chengdu, China

**Keywords:** malignant tumors, signaling pathway, miRNA, targeted therapy, antitumor molecular drugs

## Abstract

Malignant tumors remain the health problem of highest concern among people worldwide due to its high mortality and recurrence. Lung, gastric, liver, colon, and breast cancers are among the top five malignant tumors in terms of morbidity and mortality. In cancer biology, aberrant signaling pathway regulation is a prevalent theme that drives the generation, metastasis, invasion, and other processes of all malignant tumors. The Wnt/β-catenin, PI3K/AKT/mTOR, Notch and NF-kB pathways are widely concerned and signal crosstalks exist in the five solid tumors. This review provides an innovative summary of the recent progress in research on these signaling pathways, the underlying mechanism of the molecules involved in these pathways, and the important role of some miRNAs in tumor-related signaling pathways. It also presents a brief review of the antitumor molecular drugs that target these signaling pathways. This review may provide a theoretical basis for the study of the molecular biological mechanism of malignant tumors and vital information for the development of new treatment strategies with a focus on efficacy and the reduction of side effects.

## Introduction

Malignant tumors are a public health problem of worldwide concern and a leading cause of death for people in the world. According to a 2017 report by the American Cancer Society, lung cancer (LC), gastric cancer (GC), liver cancer, colorectal cancer, and breast cancer (BC) remain the top five causes of cancer deaths (Siegel et al., 2020). The highly invasive nature of cancer cells is the main cause of high cancer mortality and often leads to cancer progression and metastasis. Therefore, determining the mechanism underlying the occurrence and development of malignant tumors is of great importance.

The numerous studies that have been performed in recent decades suggest that signaling pathways are implicated in the development of cancer. Abnormal pathways drive the generation, metastasis, invasion, and other processes of all malignant tumors. Among the five tumors that received the most attention and emerged as crosstalk pathways were Wnt/β-catenin pathway, PI3K/AKT/mTOR, Notch and NF-kB pathway (Rogers et al., 2008; Ahmed et al., 2013; Collu et al., 2014; Zheng et al., 2017). Four signaling pathways play important roles in the occurrence, development and spread of malignant tumors. These pathways include the secretory glycoprotein (Wnt)/β-serial protein (β-catenin) signaling pathway (Holstein, 2012), the phosphatidylinositol-3-kinase (PI3K)/protein kinase B (AKT)/mammalian rapamycin target protein (mTOR) signaling pathway (Engelman et al., 2006), the Notch signaling pathway (Kopan and Ilagan, 2009), and the nuclear factor kappa beta (NF-kB) signaling pathway (Karin, 2006). The Wnt/β-catenin signaling pathway is engaged in cell multiplication, migration, genetic stability, and apoptosis, as well as participates in maintaining the pluripotent state of adult stem cells, which is associated with tumor generation and development (Holstein, 2012). PI3K/AKT/mTOR signal transduction pathway can not only control cell metabolism, movement, survival and proliferation, but also can affect the growth and spread of cancer cells. The PI3K/AKT/mTOR pathway dysregulation are often observed in human cancers (Engelman et al., 2006). In addition, the Notch signaling pathway has been detected in human BC, indicating that it acts an indispensable part in BC occurrence as a result of its participation in cell growth process (Kopan and Ilagan, 2009). Furthermore, the NF-κB signaling pathway has been increasingly considered to be a key factor in many steps of cancer occurrence and development (Karin, 2006). Therefore, all of these signaling pathways are frequently seen in malignant tumors. Meanwhile, an increasing number of new drugs that target these signaling pathways have been used in the gene therapy of malignant tumors (Polivka and Janku, 2014). Therefore, an improved understanding of the above signaling pathways may improve oncologists’ prognosis ability and prediction accuracy for treatment response.

MicroRNAs are a group of very conservative small single-stranded noncoding RNA molecules that, by pairing with complementary RNA molecules, negatively regulate the expression patterns of different genes at the post-transcriptional or translational level, thereby inhibiting translation through RNA degradation. MiRNAs participate in many cellular processes, such as transcription, cell growth, proliferation, inflammation, cell movement, differentiation, and apoptosis, as well as in the cell cycle (Iqbal et al., 2019). Mirna can as a tumor suppressor gene (TSG) or oncogene by regulating the expression levels of several proteins, so that the abnormal expression patterns of microRNA-related signaling pathways are associated with the occurrence and development of human malignant tumors. The miRNA-based therapies of tumors have been widely reported. Given that miRNAs are effective modulators of resistance, they could be a beneficial strategy of therapy, especially for resistant phenotypes, in conjunction with chemotherapy or radiotherapy (Yu et al., 2015; Arab et al., 2017). MiRNA-based therapeutic strategies targeting signaling pathways have been made to decrease the level of expression of specific miRNAs and supplement the deficiency in miRNAs that develops during disease progression.

This review provides an innovative summary of several signaling pathways that have been associated with the occurrence and progression of malignant tumors with high morbidity and mortality worldwide in recent years (Leake et al., 2012; Rapp et al., 2017; Stotz et al., 2015). Notably, we describe some key signaling molecules involved in the pathogenesis of malignant tumors and the drugs targeting these pathways that have been marketed (Lagadec et al., 2008; Rakha and Green, 2017). In addition, this article presents an analysis of the current research related to the role of miRNAs in cancer invasion and metastasis with the expectation that the further exploration of miRNA-targeted therapy may help establish a new spectrum of cancer treatments. The goal of our study is to help develop and design new drugs that will extend the lives of patients with the five top-ranked malignancies and reduce side effects, risks, and drug resistance.

## Targeting Wnt/β-Catenin Pathway for Cancer

### Wnt/β-Catenin Pathway

The Wnt family is a class of proteins involved in many functions, including cell survival, stem cell renewal, and organ formation (Holstein, 2012). The name Wnt is determined by the *Drosophila* gene *Wingless* and the mouse proto-oncogene *Int1* (Katoh and Katoh, 2005). At present, researchers have discovered nearly a hundred Wnt genes in different species. In humans, 19 Wnt proteins have amino acid sequence homologies of 27–83% and the conservative pattern of 23 or 24 cysteine residues. Cysteine-rich glycoproteins in the Wnt family act as ligands for up to 15 receptors or coreceptors (Li et al., 2006).

**FIGURE 1:**
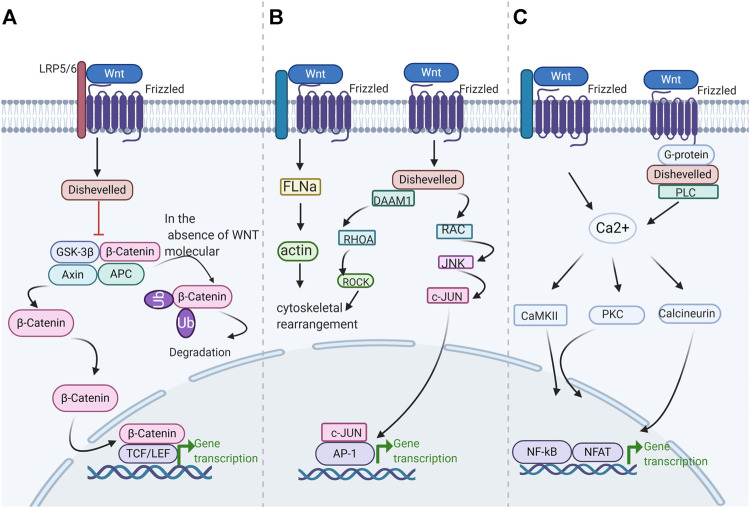
Schematic diagram of the Wnt/β-catenin pathway. Wnt signaling is transmitted through at least three different intracellular pathways **(A)** canonical Wnt/β-catenin signaling pathway **(B)** Wnt/Ca^2+^ pathway **(C)** Wnt/PCP pathway.

Wnt signals transduction through three distinct cellular pathways, including the Wnt/β-catenin-dependent or canonical pathway and the Wnt/β-catenin-independent or noncanonical pathway. The β-Catenin independent pathway includes the Wnt/Ca^2+^ pathway and the planar cell polarity (PCP) pathway. The β-catenin-dependent signaling pathway is initiated by the binding of the Wnt ligand to lipoprotein receptor-related protein (LRP)-5/6 receptors, which activate disheveled (DVL) and in turn induce the recruitment of the receptor axis inhibition protein Axin, glycogen synthase kinase-3β (GSK-3β), casein kinase 1 (CK1), and adenomatous polyposis *Escherichia coli* (APC) in the form of a complex (Macdonald et al., 2009). The Wnt-Frizzled-Axin-LRP-5/6 complex sequesters cytosolic GSK-3β, preventing it from phosphorylating β-catenin. Unphosphorylated β-Catenin accumulates in the cytosol migrating to the nucleus, where it interacts with the T cell-specific factor (TCF) lymph enhancer-binding factor and coactivators, such as pygopus and B-cell lymphoma 9, to turn on Wnt target genes, such as *c-myc*, *cyclin D1*, and cyclin-dependent kinase inhibitor 1. In the lack of the Wnt molecule, β-catenin in the cytosol is phosphorylated by GSK-3β and subsequently isolated from the β-catenin destruction complex (Wu and Pan, 2010). Phosphorylated compounds enhance β-catenin ubiquitination and lead to subsequent proteasome degradation. In the atypical Wnt pentachlorophenol or PCP pathway, the Wnt protein binds to the frizzled transmembrane receptor on the cell surface, subsequently activates RHO/RAC small GTPase and Jun N-terminal kinase (JNK) through DVL, and then assists in the regulation of the cytoskeleton and gene expression (Gordon and Nusse, 2006). DVL connects to the downstream effector ras homolog family member A and RHO-associated kinase through the DVL-associated activator of morphogenesis-1 (Fumoto and Kikuchi, 2019). In the Wnt/Ca^2+^ pathway, Wnt proteins are mainly composed of Wnt1, Wnt5a, and Wnt11. They bind to frizzled transmembrane receptors and participate in several cellular processes. These processes involve the stimulation of the heterodimer G protein to further activate phospholipase-C (PLC) (Okamoto et al., 2014). PLC increases intracellular Ca^2+^ release, decreases cGMP levels, and activates calcineurin and protein kinase-C or Ca^2+^-calmodulin-dependent protein kinase-II (Macdonald and He, 2012; Jiang et al., 2015). These processes may spur the nuclear factor of activated T cells and other transcription factors, such as cAMP response element-binding protein-1 (Jernigan et al., 2010) (Figure 1). The Wnt signaling pathway is activated in various cancers, and these molecules are closely related to its activation.

Wnt signals are activated in the crypt floor, which are crucial for cell repair and the optimal maintenance of stem cells. Numerous of evidence indicates that colorectal cancer is under the effect of Wnt signaling pathway activation, which is related to the loss-of-function of the tumor regulator APC (Krishnamurthy and Kurzrock, 2018). The Wnt pathways can cross-talk with the Notch pathway. This phenomenon provides a reliable idea for cancer treatment and intervention. Nonetheless, considerable challenges are encountered in targeting the Wnt pathway. These challenges include the search for drugs that are effective in cell repair and tissue homeostasis without impairing the functional system of normal stem cells (Yang et al., 2016; Li et al., 2018a; Xing et al., 2019). Next, we retrospect the Wnt/β-catenin pathway in different cancers. Moreover, we will introduce the status of research on the effectors and inhibitors of the Wnt/β-catenin signaling pathway.

### Wnt/β-Catenin Pathway and Lung Cancer

The abnormal Wnt/β-catenin pathway drives the generation, metastasis, invasion, and other processes of LC and plays an active role in the formation of LC angiogenesis and the stability of LC stem cells. Abnormalities in the Wnt/β-catenin pathway involve gene mutations in the Wnt pathway, molecular modifications, and protein molecular changes in transcription and translation (Ullah et al., 2013; Santana et al., 2020). Therefore, targeting the Wnt/β-catenin pathway has become an effective means to treat LC.

Given that APC is part of the degradation scaffold for β-catenin, mutations in *APC*, including cyclin D1 and c-myc, lead to decreased degradation and increased nuclear accumulation (Yedid et al., 2016). Studies have shown that loss of heterozygosity on chromosome 5q and increased levels of β-catenin at the APC locus have occurred in LC types. However, LC types are characterized mainly by transcriptional dysregulation of Wnt ligands rather than specific site mutations in the APC or β-catenin genes (Wang et al., 2019). For example, a common feature of certain LC cell lines is the loss of Wnt7a mRNA (Ohgaki et al., 2004; Nakayama et al., 2014). Elevated levels of *Wnt1* and *Wnt2* in some other NSCLCs also have been reported. The inhibition of Wnt2-induced signaling results in the down-regulation of the antiapoptotic gene and consequently initiates apoptosis (Winn et al., 2005; Chen et al., 2018). The SRY-like HMG box 2 (*Sox2*) gene codes for the SOX2 transcription factor which is expressed in the main histological types of LC (Chen et al., 2012). By inhibiting the expression of SOX2 in lung cancer, the expression of *Wnt1/2*, *Notch1* and *c-myc* genes can be down-regulated and tumor cell apoptosis can be induced (Nakatsugawa et al., 2011). By contrast, the sustained β-catenin signaling hinders the differentiation of clara cells into ciliated cells, whereas the Knockout of β-catenin in basal cells can inhibit proliferation and prompts apoptosis (Yedid et al., 2016). Besides, *Wnt7b* is up-regulated in adenocarcinoma, and *Wnt5a* is up-regulated in primary squamous cell carcinoma (Ghosh et al., 2013). In lung metastasis, the overexpression of *Wnt5a* in the noncanonical pathway also regulates the expression of fibroblast growth factor (FGF) 10 and sonic hedgehog and epithelial–mesenchymal transition (EMT). Matrix metalloproteinases are the targets of canonical and noncanonical Wnt signaling pathways and are critical for tissue remodeling; they are increased in spread tumors (Mao et al., 2018). In addition to Wnt *in vitro*, other molecules in the Wnt/β-catenin pathway are abnormally regulated in LC. For instance, the overexpression of DVL-3, a signal converter molecule and a positive regulator of the Wnt/β-catenin signaling pathway, has been reported in NSCLC. Moreover, the Wnt pathway antagonists DicKKOPF-3, WNT inhibitor (Mao et al., 2018), and secreted crimped protein-related proteins have been reported in different subtypes of LCs (Lee et al., 2004).

In addition to the genetic studies described above, epigenetic research has grown rapidly over the past period, especially for miRNAs (Lee et al., 2004). Increasingly miRNAs have been discovered that related with various types of LCs. Several β-catenin interacting proteins have also been found among the miR-214 targets in lung adenocarcinomas (Qi et al., 2015), and β-catenin itself is rather affected by miR-3619-5p. Mir-3619-5p has been reported to inhibit tumor cell growth of A549 and H460 NSCLC by binding to the 3′- UTR region of the β-catenin gene. In addition, miR-374a targets Wnt5a, and miR-487b can reduce Wnt5a activity (Qi et al., 2015)]. The type 2B sodium dependent phosphate transporter (NaPi-IIB) is situated in the apical membrane of ATII cells (Qi et al., 2015; Zhang et al., 2016). MiR-410 can activate the Wnt/β-catenin pathway by decreasing NaPi-IIB levels (Zhang et al., 2016), thus improving the ability of tumor growth and invasion.

### Wnt/β-Catenin Pathway and Liver Cancer

The inhibitors of the Wnt/β-catenin signaling pathway might be effective in the hepatocellular carcinoma (HCC) intervention therapy (Taciak et al., 2018). In a HepG2 cell line, the knockout of β-catenin under the mediation of RNA interference decreases proliferation and growth *in vitro*. Evidence showing that miRNAs correlated with the expression of Wnt/β-catenin pathway related genes, such as *c-myc*, *APC*, *cyclin D1*, and DKK1, are increasing. Ashmawy held that miR-106b, miR-10a, miR-99a, miR-148a, miR-215, miR-199a, miR-30e, miR-199a3p, miR-24, miR-122, and miR-125b, are down-regulated in patients with HCC (Ashmawy et al., 2017). Liu thought that miR-18a expression is up-regulated relative to that in adjacent nontumoral liver tissue in human HCC. Up-regulation of miR-18a expression level improved the spread and migration ability of HCC cell lines by suppressing krüppel-like factor4, a factor that negatively regulates *β*-*catenin* expression. Other than miR-18a, Liu also concluded that upon induction by *c-myc* silencing, miR-320a expression levels were upregulated in HCC tissues relative to paired adjacent non tumorous liver tissues, and the ability to inhibit HCC cell proliferation and invasion would be enhanced (Lu et al., 2017; Xie et al., 2017). These data suggest that miR-320a may be exploited as a target for HCC therapy. Moreover, wnt3 is a target of miR-1247-5p, and its expression is significantly down-regulated by miR-1247-5p overexpression. This restrains the invasion and proliferation of HepG2 cells, induces cell apoptosis *in vitro* (Chu et al., 2017). Thus, miR-1247-5p may serve as a target for HCC therapy (Table 1).

**TABLE 1:** miRNAs regulate Wnt/β-catenin pathway in liver cancer.

miRNA	Regulation	Pathway	References
miR-18a	Upregulating and promoting the proliferation and migration of HCC cell lines by inhibiting KLF4	Wnt/β-catenin pathway	Lu et al. (2017)
miR-320a	Inhibiting it can up-regulation of the expression levels of *β-catenin, c-myc, cyclin D1* and *DKK-1*	Wnt/β-catenin pathway	Xie et al. (2017)
miR-1247-5p	Inhibiting the invasion and proliferation of HepG2 cells by targeting *Wnt3*	Wnt/β-catenin pathway	Chu et al. (2017)

### Wnt/β-Catenin Pathway and CRC

Wnt/β-catenin pathway dysregulation also occurs frequently in CRC. The abnormal activation of this pathway is related to cell proliferation, invasion behavior, and drug resistance, suggesting the potential value of targeting Wnt/β-catenin pathway as an intervention method for CRC. Most CRC patients have at least one mutation in a Wnt signaling cascade gene such as *APC* and *β-catenin* protein. Gene mutations in *β-catenin*, *GSK-3β*, *Axin*, or *APC* leads to abnormal activation of the Wnt/β-catenin pathway, and simultaneous Wnt overexpression causes abnormal activation of this pathway (Schmalhofer et al., 2009). The Wnt/β-catenin pathway regulates *E-cadherin* by enhancing the expression level of repressors of this adhesion molecule, involving the transcriptional factors recombinant snail homolog (*SNAI*) *1*, zinc finger E-box binding homeobox 1 and *SNAI2*. This action therefore leads to metastasis and invasiveness.

Abnormal expression of miRNA is related to the disorder of cancer-related signaling pathways, Wnt/β-catenin pathway is included. Further research showed that Smad7 is a protein necessary for nuclear accumulation of β-catenin. MiR-93 inhibits the Wnt/β-catenin pathway by targeting Smad7 (Tang et al., 2015). Similarly, miR-185 (Dong-Xu et al., 2015) and miR-320a expression levels in CRC cells are significantly down-regulated compared with those in normal colon cells. Consistently, the enforced expression of miR-101 (Strillacci et al., 2009) attenuates the promalignant features of CRC, including cell growth, invasion and hypoxic survival, which means that miR-101 can be an effective cancer suppressor for CRC patients. Moreover, several agents targeting this pathway have been developed for CRC treatment (Table 2). These agents include retinoic acid and vitamin D. Retinoic acid can suppress Wnt signaling via interaction with β-catenin or via competing with TCF. The active form of vitamin D encourages β-catenin binding to the vitamin D receptor, and reducing the level of β-catenin. Besides, several monomers of Chinese traditional herbs, including quercetin and resveratrol, and the green tea polyphenol epigallocathechin-3-gallate are observed to inhibit Wnt inhibitory activity (Roa et al., 2019). The inhibitors of Wnt production are a new type of Porcupine-targeted Wnt antagonists. For example, LGK974 can bind to and block porcupine enzymes. LGK974 inhibits the expansion of the murine tumor xenograft model via ectopic Wnt1 expression originating from the mouse mammary tumor virus (MMTV) (Liu et al., 2013). PRI-724, a second generation β-catenin antagonist, increases p300/β-catenin binding and stem-cell differentiation. BBI608 is another small molecule that not only inhibits the signal transducer and activator of transcription-3 but also suppresses β-catenin signaling to treat colorectal cancer (Lenz and Kahn, 2014). BBI608 can be combined with chemotherapy agents (such as cisplatin, gemcitabine, paclitaxel, temozolomide, sorafenib and pemetrexed) to treat patients with CRC. However, when BBI608 was used in the phase III trial of metastatic CRC, its expected efficacy was not achieved in the short-term analysis (Ciombor et al., 2015).

**TABLE 2:** Some agents target Wnt/β-catenin pathway in colon cancer.

Agents	Regulation	Reference
Retinoic acid	Inhibit Wnt signaling by direct interaction with β-catenin/competition for TCF binding	Roa et al. (2019)
Vitamin D	Encourages the β-catenin binding to the vitamin D receptor and decreases the amount of β-catenin	Roa et al. (2019)
Quercetin	Suppresses Wnt inhibiting activity	Roa et al. (2019)
Resveratrol	Suppresses Wnt inhibiting activity	Roa et al. (2019)
Green tea polyphenol epigallocathechin-3-gallate	Suppresses Wnt inhibiting activity	Roa et al. (2019)
LGK974	Binds and blocks the porcupine enzyme	Liu et al. (2013)
PRI-724	Increase p300/β-catenin binding and stem-cell differentiation	Lenz and Kahn (2014)
BBI608	Not only inhibits signal transducer and activator of Stat3 but also suppresses β-catenin signaling	Ciombor et al. (2015)

## Targeting the Phosphatidylinositol-3-Kinase/Protein Kinase B/Mammalian Rapamycin Target Protein Pathway for Cancer

### Phosphatidylinositol-3-Kinase/Protein Kinase B/Mammalian Rapamycin Target Protein Pathway

**FIGURE 2:**
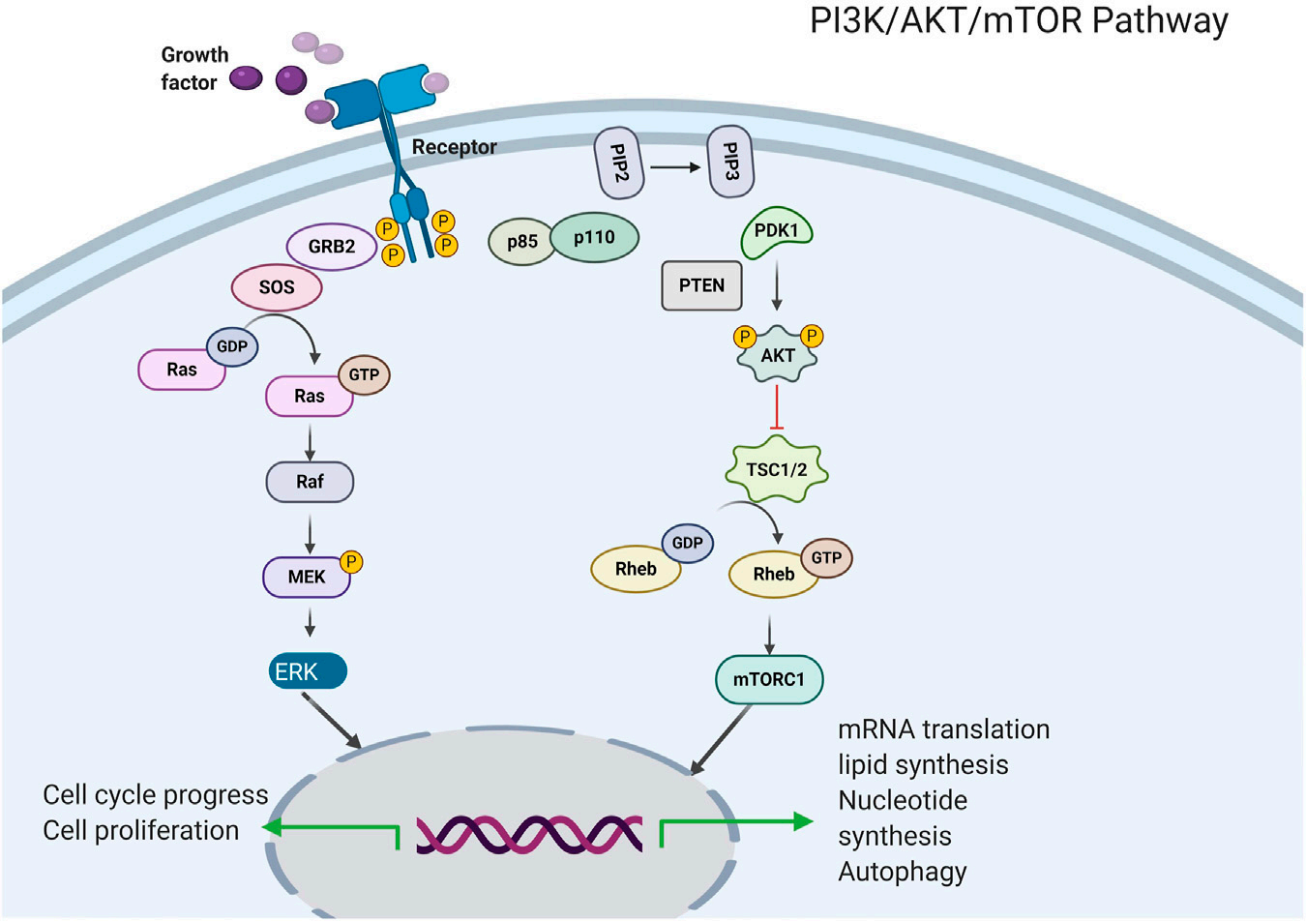
Intracellular signaling via the PI3K/AKT/mTOR pathway.

The PI3K enzyme mainly participates in the phosphorylation of the inositol lipid membrane and mediates signal transduction (Figure 2, Costa et al., 2018). Two receptor tyrosine kinases (RTKs) and non-RTKs lead to the activation of PI3K, consequently leading to the formation of a second messenger, phosphatidylinositol 3, 5-triphosphate, from phosphatidylinositol 4, 5-diphosphate. PI3K activation recruits pleckstrin homology domain-containing proteins, including AKT/PKB kinases (Engelman et al., 2006) to the cell membrane, therefore driving conformational changes and resulting in phosphorylation at threonine 308 and at serine 473. which via the active phosphoinositide-dependent kinase 1 and via phosphoinositide-dependent kinase 2, respectively. Activated AKT kinases are capable of phosphorylating tuberous sclerosis protein 1 (TSC1) and tuberous sclerosis protein 2 (TSC2) (Kwiatkowski et al., 2016). In addition, the activity of the kinase mTOR is negatively regulated by the TSC1/TSC2 complex, therefore, AKT leads to that the mTOR complex 1 (mTORC1) are activated, this effect ultimately leads to lipid synthesis and increased protein and decreased autophagy, thereby supporting cell expansion and cell development (Cheng et al., 2015). Particularly, mTORC1 participates in a negative feedback route that prevents AKT over-activation. The PI3K/AKT/mTOR pathway could be up-regulated via the activation of molecular alterations in AKT subunits, PI3K, and mTOR or via inhibiting the PI3K regulatory subunit gene of phosphate and tension homology deleted on chromosome ten (PTEN), TSC1, TSC2, and serine/threonine protein kinase LKB1 (Moulder et al., 2015).

The therapeutic targeting of the PI3K/AKT/mTOR pathway has led to the advancement of some different kinds of drugs, PI3K and AKT inhibitors, as well as catalytic and allosteric mTOR kinase inhibitors are included. For example, the mTOR inhibitors Temsiro-Limus and Everolimus and the PI3K inhibitors Idelalisib and Copanlisib have been accepted by the FDA for Clinical treatment of patients with cancer (Janku et al., 2011; Moulder et al., 2011; Janku et al., 2014). However, many questions related to the inhibitors of the PI3K/AKT/mTOR pathway remain unanswered. They include which drugs or kinds of drugs are ought to be used in a specific cancer environment and whether the formulation of a reasonable combination strategy will improve the efficacy of tumor treatment.

### Phosphatidylinositol-3-Kinase/Protein Kinase B/Mammalian Rapamycin Target Protein and Lung Cancer

Dysregulated PI3K/AKT/mTOR pathway also contributes to LC initiation and progression, and eukaryotic translation initiation factor 4E (eIF4E), one of its downstream effectors (Mamane et al., 2004), has been recognized to function as an oncogene in many studies. It is not only able to transform cells that are overexpressed in adenocarcinoma, but also can cause poor prognosis factors. Increasing evidence supports that the mTOR pathway plays a part in lung carcinogenesis by binding to eIF4E. The mTOR pathway is involved in the occurrence and development of lung cancer. Negative regulators of mTOR signaling, such as LKB1 and PTEN, are frequently mutated in LC and are considered tumor suppressors (Phillips et al., 2005). Studies have shown that the mTOR signaling pathway act a significant role in behaviors such as aggressiveness and metastasis of LC. Exposure of NSCLC cells to epidermal growth factors or hypoxia causes the activation of hypoxia inducible factor-1 (HIF-1), which ultimately leads to the significant upregulation of C-X-C motif chemokine receptor 4 (CXCR4) expressions and chemotactic behavior. Fortunately, activation of HIF-1 α was inhibited by mTOR inhibitor rapamycin as well as PI3K inhibitors wortmannin and LY294002, and consequently upregulation of CXCR4 expression was inhibited (Sarkaria et al., 2007).

MTOR pathway agents become tempting targets for LC treatment. For example, many models *in vitro* and *in vivo* have demonstrated the antiproliferative and antitumor effects of rapamycin (Phillips et al., 2005). CCI-779 is a water-soluble ester of sirolimus, whereas everolimus is an oral sirolimus analogue, and studies have suggested that both could be used to treat lung cancer. And is used for the advanced renal cell carcinoma therapy (Pandya et al., 2007). PI3K inhibitors have also emerged as effective agents to inhibit the mTOR pathway. Although many PI3K inhibitors exhibit preclinical antitumor activity, they fail to achieve estimated efficacy at the time of clinical evaluation. For example, the PI3K inhibitors wortmannin and LY2994002 have been proven to be unable for clinical treatment in spite of preclinical anticancer ability. Novel PI3K inhibitors have been developed for the treatment of LC and other solid tumors, including BKM120, XL147, and GDC-0941, are ongoing, data regarding LC remain pending (Ekman et al., 2012, Table 3).

**TABLE 3:** PI3K/AKT/mTOR inhibitors in cancers.

Compound	Target	Cancer	Clinical symptoms	Reference
Wortmannin	PI3K	Lung cancer and breast cancer and other solid tumors	Poor solubility, instability, and high toxicity	Ekman et al. (2012)
LY2994002	PI3K	Lung cancer and breast cancer and other solid tumors	Poor solubility, instability, and high toxicity	Ekman et al. (2012)
BKM120	PI3K	Gastric cancer	Well tolerated, high toxicity	Yang et al. (2018b)
PX-886	PI3K	Gastric cancer	Instability, and high toxicity	Yang et al. (2018a)
XL147	PI3K	Gastric cancer	Poor solubility, instability, and high toxicity	Gravina et al. (2016)
WX-037	PI3K	Gastric cancer		Haagensen et al. (2016)
BYL719	PI3K	Gastric cancer	Poor solubility, instability, and high toxicity	Juric et al. (2018)
GDC0032	PI3K	Gastric cancer		Juric et al. (2018)
P7170	PI3K/mTOR	Gastric cancer, lung cancer		Jalota-Badhwar et al. (2015)
BEZ235	PI3K/mTOR	Gastric cancer	Well tolerated, gastrointestinal toxicity	Kim et al. (2019)
XL765	PI3K/mTOR	Gastric cancer		Gravina et al. (2016)
GDC-0980	PI3K/mTOR	Gastric cancer, breast cancer	Poor solubility, instability, and high toxicity	Kim et al. (2019)
GDC-0941	PI3K/mTOR	Gastric cancer	Poor solubility, instability, and high toxicity	Ekman et al. (2012)
SF1126	PI3K/mTOR	Gastric cancer	Poor solubility, Instability, and high toxicity	Kim et al. (2019)
PF-05212384	PI3K/mTOR	Gastric cancer		Kim et al. (2019)
PF-4691502	PI3K/mTOR	Gastric cancer		Kim et al. (2019)
VS-558	PI3K/mTOR	Gastric cancer		Kim et al. (2019)
MK-2206	Allosteric AKT	Gastric cancer	Well tolerated, high toxicity	Hirai et al. (2010)
AZD5363	Catalytic AKT	Gastric cancer		Brown and Banerji (2017)
GSK690693	Catalytic AKT	Gastric cancer	Poor solubility, instability, and high toxicity	Brown and Banerji (2017)
Everolimus	mTOR	Breast cancer	Tends to have an infection, including bacterial, fungal, and viral infections, as well as reactivation of hepatitis B virus/increased incidence of fatigue, asthenia, and anorexia	Janku et al. (2014)

### Phosphatidylinositol-3-Kinase/Protein Kinase B/Mammalian Rapamycin Target Protein and Gastric Cancer

The molecular characterization of gastric cancers has revealed that PI3K/AKT/mTOR pathway abnormalities are associated with a high recurrence rate of gastric cancer, suggesting that these molecules are potential therapeutic targets (2014). Aberrant PI3K pathway activation is mediated by mechanisms including altered genes for AKT and PIK3CA, decreased upstream RTK, *PTEN* expression, and other less frequent events. *PIK3CA* with mutations and amplification has been seen frequently in gastric cancer. *PTEN* negatively regulates PI3K activity under physiological conditions. It is a TSG located on chromosome 10q23.3 (Markman et al., 2010). The *Akt* family of genes consists of the *AKT1*, *AKT2*, and *AKT3* genes. Different isoform gene expression leads to difference in function. For example, *AKT1* enhances cellular survival and proliferation ability, the loss of *AKT1* can increase invasiveness. Paradoxically, *AKT2* promotes mesenchymal transformation and cellular invasive behavior, and the loss of *AKT2* expression might weaken ability of metastatic (Cariaga-Martinez et al., 2013).

Consequently, four major kinds of drugs that target the PI3K/AKT/mTOR pathway have been identified (Table 3): PI3K, AKT, PI3K/mTOR, and mTOR inhibitors. The PI3K inhibitors BKM120 (Yang et al., 2018a), PX-886 (Yang et al., 2018b), XL147 (Gravina et al., 2016), WX-037 (Haagensen et al., 2016), BYL719 (Juric et al., 2018), and GDC0032 (Juric et al., 2018) were designed treat gastric cancer. PI3K/mTOR inhibitors include P7170 (Jalota-Badhwar et al., 2015), BEZ235 (Kim et al., 2019), XL765 (Gravina et al., 2016), GDC-0980 (Kim et al., 2019), SF1126 (Kim et al., 2019), PF-05212384 (Kim et al., 2019), PF-4691502 (Kim et al., 2019), and VS-558 (Kim et al., 2019). AKT inhibitors include MK-2206 (Hirai et al., 2010), AZD5363, and GSK690693 (Brown and Banerji, 2017). Although PI3K signaling inhibitors appear promising, several theoretical shortcomings raise concerns regarding their clinical efficacy (Engelman, 2009). PI3K inhibitors exhibit obvious antitumor effect when used alone. Unfortunately, relevant studies have shown that gastric cancer may become resistance to combination therapy, so combination therapy with PI3K signaling pathway inhibitors is unlikely to be useful for gastric cancer.

### Phosphatidylinositol-3-Kinase/Protein Kinase B/Mammalian Rapamycin Target Protein and Breast Cancer

Recent life science studies have proposed that aberrant PI3K/AKT/mTOR signaling pathway activation is the key to the shortened life cycle of cancer patients and that aberrant activation of this pathway is a vital mechanism of resistance to targeted therapies (Droog et al., 2013). It is estimated that aberrant activation of the mTOR pathway occurs in 70% of all BC cases. In BC, the genes that encode several process of the mTOR pathway are altered. On the one hand, mutations or amplifications of some genes become activated, including those encoding insulin-like growth factor 1 receptor, PIK3CA, RAC-α, AKT1, and human epidermal growth factor receptor 2 (HER2). On the other hand, the expression of genes encoding PTEN and LKB156 are decreased or even no function (Wu et al., 2015; Luey and May, 2016). Breast cancer growth depends on a certain level of estrogen; this conclusion is because nearly 75% of BC cases are ER+. The mechanism of selective estrogen receptor modulators in the treatment of patients with ER + BC is to block the binding of nuclear ERα to estrogen by binding to nuclear ERα, thereby blocking receptor activation. Tamoxifen is such a drug. However, the problem of resistance to the drugs is still widespread in patients with ER + BC (Droog et al., 2013). MTOR pathway is the primary factor of drug resistance in ER + BC patients. This is because the estrogen-independent estrogen receptor (ER) transcriptional activity is activated by this pathway, making the ER highly sensitive to activation, so that the possibility of tamoxifen binding to nuclear ERα was reduced. HER2 expression is closely correlated with enhanced aggressiveness and significantly worse prognosis in BC and it is a key biomarker for BC. This is because mTOR signaling can be activated by HER family receptors, in particular, HER2 expression is critical for over activation of mTOR pathway in BC. MTOR signaling is associated with tolerance to HER2 therapeutic mechanisms in BC, the case in point is the dual epidermal growth factor receptors HER1 and HER2, the antibody-based agent trastuzumab, and the inhibitor lapatinib (Luey and May, 2016).

Apamycin was the first available mTOR inhibitor for BC therapy and was originally developed as an immunosuppressant for transplant recipients. Researchers classified the new generation of mTOR inhibitors into PI3K/mTOR inhibitors and mTORC1/2 inhibitors. Besides, more and more compounds that block the upstream of the mTOR pathway have been developed. These compounds include AKT and PI3K inhibitors (Table 3). LY294002 and Wortmannin are the earliest and most widely studied PI3K pathway inhibitors (Verret et al., 2019). BC cell model experiments have proved that the inhibitors have strong anti-tumor effects, and also confirmed that they can inhibit the PI3K pathway, but unfortunately they have the disadvantages of poor solubility, instability and high toxicity, so they are limited to pre-clinical studies (Fruman et al., 2017; Liu et al., 2017). In the future research, the main issues need to be paid attention to include whether a relational treatment combination can completely block or partially inhibit mTOR pathway, and whether subtype specific or pan-PI3K inhibition can provide additional benefits for BC patients.

### Phosphatidylinositol-3-Kinase/Protein Kinase B/Mammalian Rapamycin Target Protein Pathway and Liver Cancer

In cell lines, miR-758-3P repair inhibits cell proliferation, migration, and invasion. Jiang and his colleagues showed that miR-758-3P significantly down-regulates the expression of murine double minute 2 (MDM2) and mTOR while up-regulating the expression of p53, AKT, and PRAS40 (Jiang et al., 2017). In addition, effector AKT can regulate downstream mTOR and inhibits PRAS40, thus eliminating the inhibitory effect on mTORC1. IGF-1R can regulate behaviors such as cell progress, migration and invasion in HCC and is activated via the mTOR pathway. MiR-187 (Han et al., 2017), miR-497 (Cheng et al., 2017), miR-99a (Han et al., 2017) and miR-592 (Wang et al., 2017) are targeted IGF1R. Han found that miR-187 is down-regulated in HCC tissues and cell lines and reported that the recovery of miR-187 will lead to a significant halt in the growth of HCC (Han et al., 2017). Moreover, miR-497 and miR-99a could target not only IGF-1R but also 3′-UTR of mTOR, and their down-regulation was observed in HCC human tissues and cell lines (Cheng et al., 2017). Wang confirmed that miR-592 was lowly expressed in HCC cell lines, and affected metastasis to lymph nodes (Wang et al., 2017). It has been demonstrated that miR-2965p inhibits HCC cell proliferation, migration and invasion through targeting AKT2 (Ma et al., 2017). These findings show that the miR-187, miR-497, miR-99a, miR-592 and miR2965p can be an effective target for HCC treatment. Yu showed that miR-142 can directly target transforming growth factor β (TGF-β), and mTOR is one of the effector pathways of TGF-β signaling. Additionally, TGF-β signaling also controls cell viability, growth, EMT, and neoangiogenesis. On the whole, miR-142 can act as a TSG in HCC, being able to increase TGF-β-induced HCC development (Yu et al., 2017). Moreover, miR-23b has been demonstrated to regulate ST7L, a suppressor of the AKT/GSK-3β/β-catenin signaling in HCC cells (Jiang et al., 2017). MiR-181A (Han et al., 2017), miR-155-5p (Cheng et al., 2017), and miR-25 (Wang et al., 2017) are up-regulated in HCC tissues. HCC plays a carcinogenic part in HCC through targeting PTEN (Table 4).

**TABLE 4:** MicroRNAs regulate PI3K/AKT/mTOR pathway in liver cancer.

miRNA	Regulation	Pathway	References
miR-758-3P	Down-regulated the expression of MDM2 and mTOR/Upregulated the expression of p53, AKT and PRAS40	PI3K/AKT/mTOR pathway	Jiang et al. (2017)
miR-187	Leads to a significant halt in the growth of HCC.	PI3K/AKT/mTOR pathway	Han et al. (2017)
miR-497	Target the 3′-UTR of IGF-1R and mTOR, decrease tumor proliferation and tumor growth	PI3K/AKT/mTOR pathway	Cheng et al. (2017)
miR-99a	Target the 3′-UTR of IGF-1R and mTOR, decrease tumor proliferation and tumor growth	PI3K/AKT/mTOR pathway	Han et al. (2017)
miR-592	Down-regulated in HCC tissues and cell lines, and was associated with lymph node metastasis	PI3K/AKT/mTOR pathway	Wang et al. (2017)
miR2965p	Inhibited HCC cell proliferation, migration and invasion *in vitro* by targeting AKT2	PI3K/AKT/mTOR pathway	Yu et al. (2017)
miR-142	Controls cell vitality, proliferation, (EMT) and neo-angiogenesis target TGF-β	PI3K/AKT/mTOR pathway	Yu et al. (2017)
miR-23b	As a suppressor of the AKT/GSK3β/β-catenin pathway in HCC cells by regulating ST7L	PI3K/AKT/mTOR pathway	Jiang et al. (2017)
miR-181A	Plays a carcinogenic role targeting PTEN	PI3K/AKT/mTOR pathway	Han et al. (2017)
miR-155-5p	Plays a carcinogenic role targeting PTEN	PI3K/AKT/mTOR pathway	Cheng et al. (2017)
miR-25	Plays a carcinogenic role targeting PTEN	PI3K/AKT/mTOR pathway	Wang et al. (2017)

## Targeting the Notch Pathway for Cancer

### Notch Pathway

**FIGURE 3:**
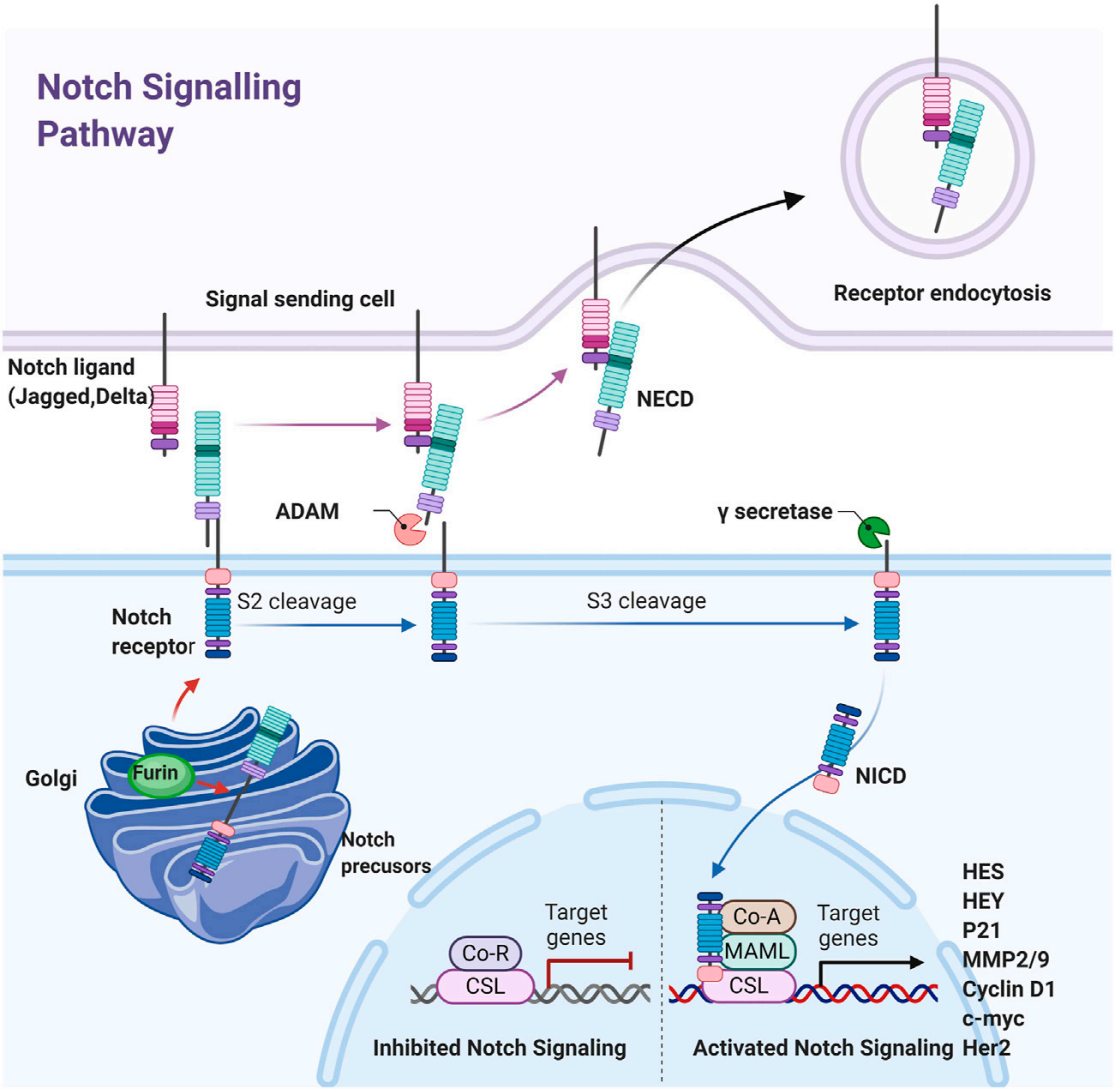
Schematic diagram of the Notch pathway. The Notched mono-precursors are furin-cut in Golgi bodies to form mature Notched receptors and transferred to the plasma membrane. The Notch is activated when Notch ligands on adjacent cells combined with them, leading to second and third cut by ADAM and γ-secretes, releasing Notch intracellular domain NICD, which is transferred into the nucleus and combined with CSL to initiate downstream gene expression.

The Notch signaling pathway is evolutionarily conservative and was originally thought to have a vital role in all kinds of developmental processes (Zardawi et al., 2010; Danza et al., 2013). It is found in the gap that appears on fruit fly wings and acts an indispensable role in embryonic development (Ai et al., 2012). The signaling pathway is composed of these parts: Notch receptor, Notch ligand, and the DNA-binding sequence CSL (CBF1/Su(H)/lag-1. The Notch pathway in humans possesses five ligands (*Jagged-1*, *Jagged-2*, *DLL-1*, *DLL-3*, and *DLL-4*) and four receptors (*Notch-1*, *Notch-2*, *Notch-3*, and *Notch-4*) (Reichrath and Reichrath, 2020). The Notch ligand is also a type I transmembrane protein that contains extracellular repeat sequences similar to the EGF, a Delta/serrate/lag2 motif that accounts for Notch interplay, and short and highly dispersed intracellular domains. When a ligand combine with Notch receptors between two nearby cells, the Notch signaling pathway is activated to control cell growth and regulate organogenesis and morphogenesis (Figure 3). Total three steps of proteolytic cleavage are required in canonical Notch activation process (Teodorczyk and Schmidt, 2014). First, the Notch single-chain precursor is cut by Furin protease in the Golgi complex to form large fragments containing extracellular domains and small fragments containing intracellular and transmembrane regions. Mature heterodimer receptors are formed through Ca^2+^-dependent noncovalent bonding and transferred to the cell membrane. When the mature receptor binds to a ligand, it performs a second cleavage with TACE or a member of the ADAM de-integrin and metalloproteinase family to release extracellular fragments. The remaining fragment, which consists of transmembrane and intracellular regions, is dissected for the third time by γ-secretase for the release of the soluble Notch intracellular region (NICD) and transfer to the nucleus (Suresh and Irvine, 2015). CSL is bound with NCID and lead to specific gene expression. The Notch signaling pathway exhibits CSL nondependent activation in addition to CSL-dependent activation. However, a great majority Notch target genes exist CSL binding sites. The signal transduction receives signals from neighboring cells and transfers them to the nucleus, thus spurring the expression of transcription factors (Uzhachenko and Shanker, 2016) (Figure 3).

Although Notch signaling does not amplify signals, the cell differentiation is precisely controlled (Kopan and Ilagan, 2009). Notch target genes include members of the HES and HEY families, *MMP-2*, *MMP-9*, *cyclin D1*, *Her2*, *c-myc*, and apoptosis-related genes (Wu et al., 2010; Ranganathan et al., 2011; Li et al., 2014; De Francesco et al., 2018; Krishna et al., 2019). The Notch pathway may regulate cell growth, differentiation, and tumor metastasis exactly through regulating the expression of these genes (Zardawi et al., 2009; Zhou et al., 2013). Dysregulated Notch signaling due to mutations, amplification, or ligand and/or receptor over-expression is associated with many malignancies. Given that Notch signaling plays a critical role in cancer genesis and prognosis, it is a promising approach for cancer therapy to block Notch pathway.

### Notch Pathway and BC

Notch has been identified as an oncogene in mice infected with MMTV in the past few years. It has also been detected in human BC and likely plays a significant part in BC development (Reichrath and Reichrath, 2020). The American Cancer Association states that BC can be classified into four BC molecular subtypes with prognostic differences in terms of patient outcome on the basis of the presence or absence of the expression of specific biological markers: ERs, progesterone receptors, and HER2. The Notch system, in cooperation with the VEGF pathway, engages in the adjusting of angiogenesis and sprouting in BC (Zhang et al., 2019). Notch expression is controlled by hypoxia and inflammatory cytokines (IL-1, IL-6, and leptin). Endothelial cells can develop into tip or stalk cellular norm under pro-angiogenic signal (Zhang et al., 2019). The acquisition of the high expression of Notch ligands is indisputably related to the aggressive clinical behavior of tumors. BC clinical symptom is accompanied by the high expression of the *Notch-1*, *Notch-3*, and *Notch-4* pathways, and *Notch-2* has been considered to be a tumor suppressor in previous research (Shen and Reedijk, 2021). The evidence of the essential role of the Notch system in BC originates from MMTV, wherein *Notch-1* and *Notch-4* genes have been detected. The genes *Notch-1* and *Notch-4* are the major targets of MMTV. Mutations that induce epithelial mammary oncogenesis are created through insertion and rearrangement (Guo et al., 2011).

In view of the key role of Notch in BC cell proliferation, differentiation, invasion, and drug resistance, this pathway has become a potential target for BC prevention and treatment. Notch targeting agents have been widely used in clinical trials, including γ-secretase inhibitors and monoclonal antibodies. γ-secretase inhibitors are small agent that used frequently (Suresh and Irvine, 2015; Uzhachenko and Shanker, 2016). GSI is one of γ-secretase inhibitors, which achieves anti-tumor effects by reducing the levels of activated Notch and several other substrate proteins in cells. GSI MK-0752 has been proven to have a good anti-BC effect in clinical trials (Krishna et al., 2019). Unfortunately, continuous use of GSI drugs can cause serious side effects to the body of BC patients. Therefore, GSI drugs are generally not used alone but combined with chemotherapy to treat BC. Monoclonal antibody is also a biological drug that can effectively resist BC, For example, trastuzumab and pertuzumab have been successfully used to treat patients with metastatic BC overexpressing HER2 by targeting Notch receptors (Shen and Reedijk, 2021).

## Targeting the NF-κB Pathway for Cancer

### NF-κB Pathway

The NF-κB signaling pathway in cancer has been studied for decades. The abnormal activation of NF-κB transcription factors is frequently seen in various solid tumors, such as CRC and gastric cancer (Lawrence, 2009). NF-κB pathway members and their regulatory genes response control cancer cell blood vessel formation, growth, metastasis, and tolerance to drug (Mitchell et al., 2016).

NF-κB is a Rel family transcription factor composed of RelA (p65), RelB, Rel (c-Rel), NF-κB1 (p50/p105), and NF-κB2 (p52/p100) (Oeckinghaus et al., 2011). There are three pathways of NF-κB signal transduction. NF-κB signaling could be activated by canonical and alternate pathways via an IκB kinase (IKK)-dependent manner. In the typical pathway, after activating β-subunit of IKK, the negative regulator of the NF-κB inhibitor of kappa B-α (IκBα) protein are phosphorylated and subsequent results in the ubiquitination and proteasome-mediated degradation of IκBα (Baud and Jacque, 2008; Perkins, 2012). The event results in the release of the p65/p50 heterodimer and makes the translocation of the NF-κB complex into the nucleus. The alternative pathway results in the specific activation of p52: RelB heterodimers and is not required for the activation of the highly ubiquitous p50: RelA dimers. The alternative pathway is distinguished from the classical pathway on the basis of IKK-α homodimers rather than reliance on IKK-γ and IKK-β activity, the preferred substrate of which is the precursor of p52-p100/NF-κB2. The activation of IKK-α dimers results in the degradation of the latter and the nuclear entry of p52: RelB dimers (Gilmore, 2006). Another pathway that can make NF-κB activation is based on the activation of casein kinase 2, which can phosphorylate carboxyl-terminal sites and thereby make IκBα degradation, independently of IKK. This pathway is activated only when the skin is exposed to carcinogenic ultraviolet radiation (Karin, 2006, Figure 4).

**FIGURE 4:**
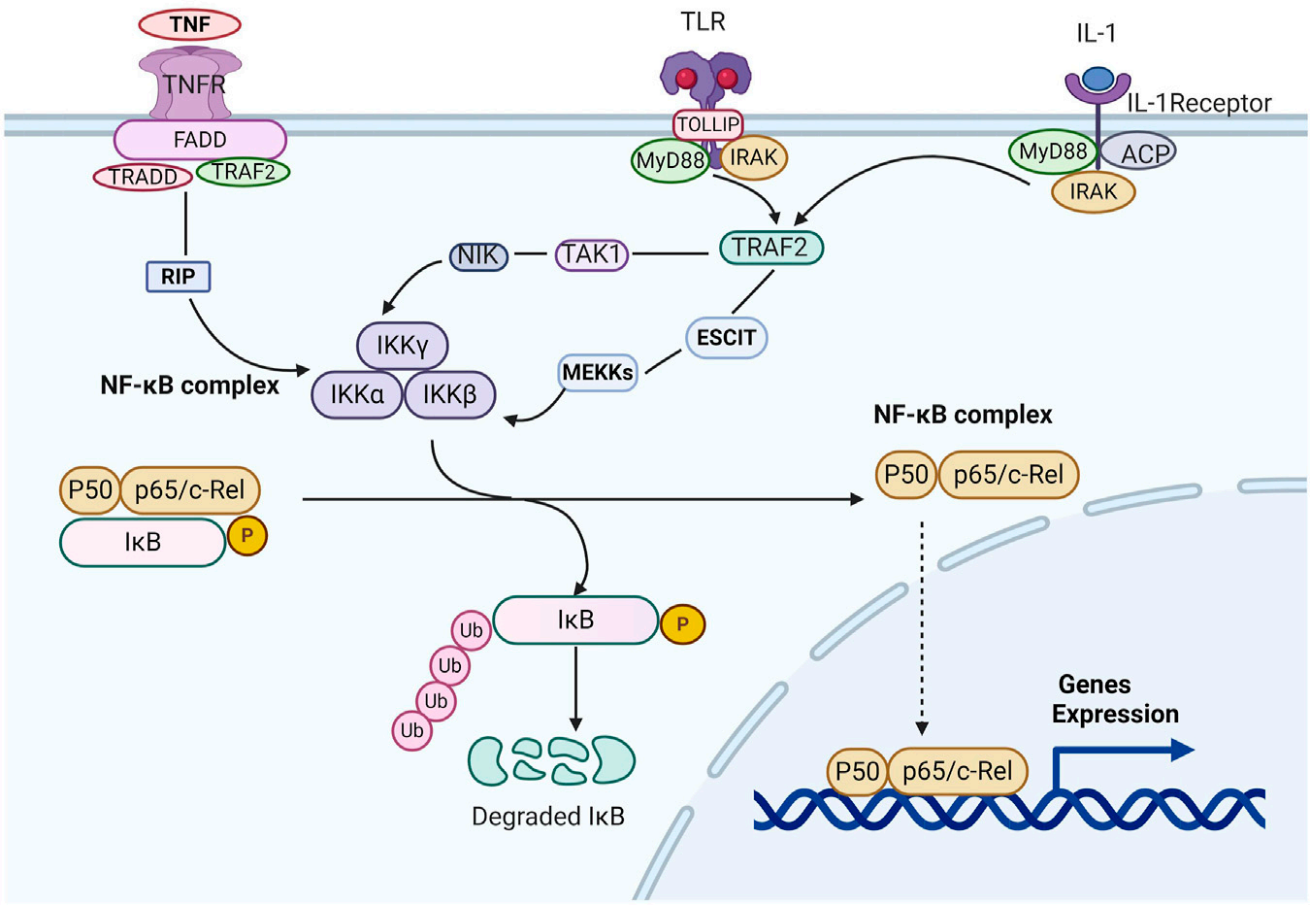
Schematic diagram of the NF-κB pathway. When the inflammatory factors such as tumor necrosis factor A/inteeleukin-1/Toll-like Receptors combine with the related receptors, they cause the configuration changes of the latter receptors, like RIP, NIK, or MEKKs. Then IKKs are activated, which can phosphorylate IκBα, and ubiquitination under the action of the ubiquitin ligase p-trcp. The ubiquitin ligase p-trcp can be recognized and degraded by 26S proteasome. Therefore, NF-κB can be released from the cytoplasm of NF-κB/IkBα complex, activate and expose the activate site domain, and rapidly transfer nucleus. Through P50 subunit binding with target genes, the expression of target genes such as TNF-α,IL-1.

NF-κB is an activator of antiapoptotic genes. It could also monitor tumor angiogenesis and metastatic invasiveness, the signaling pathways that mediate its activation have shed new light on chemotherapy and prevention of cancer (Didonato et al., 2012).

### NF-κB Pathway and CRC

NF-κB is related to angiogenesis and EMT formation in CRC and controls the CRC cells to migrate and invade. Angiogenesis represents the occurrence of cancer. Vascular endothelial growth factor (VEGF) drives the formation of some angiogenesis regulators, which include the angiogenesis regulator cyclooxygenase-2, CXCL1, CXCL8, and IL-8 in the NF-κB pathway. Monoclonal antibodies against VEGF are widely exploited for CRC therapy. VEGF is also inducible under HIF (D’ignazio et al., 2016). The HIF pathway regulates immune responses through cross-talk with NF-κB (Kwon et al., 2010). This cross-talk was proposed by numerous scholars and also proved that HIF-1α is inhibited by the NF-κB inhibitor parthenolide, which results in the down-regulation of hypoxia-dependent angiogenesis in human umbilical vein endothelial cells (Thiery et al., 2009). Moreover, in CRC tissues, histological evidence indicates that the expression of NF-κB (p65) is correlated with the expression of HIF-1α, VEGF, and vascular invasion. The role of NF-κB in the angiogenesis of CRC and its interactions with other signaling pathways provide important insights into the targeted therapy of CRC (Chu et al., 2012). Furthermore, NF-κB participates in the EMT of tumor cells. EMT leads to epithelial mesenchymal cells invasion and metastasis and is thus significant in advancing CRC metastasis. Matrix metalloproteinases (MMPs) are proteolytic enzymes that are capable of degrading the extracellular matrix and promoting tumor invasion. MMP is overexpressed in CRC and is involved in the poor prognosis and spread of CRC. The connection of mechanism between NF-κB and MMP-9 was studied in consideration of the absence of the β subunit of the IKK complex in CRC cells (Fukuyama et al., 2007). MMP-9 gene expression requires the involvement of NF-κB (p65 and IKK activity). The NIK- and IKK-β-binding protein (NIBP) has taken part in the regulation of cytokine-induced canonical NF-κB signaling. Activin A is overexpressed in human CRC, particularly tumors in stage IV disease, implying that activin A might play a part in advanced CRC (Patel et al., 2018; Soleimani et al., 2020). Related reports have pointed out that activin A induces NF-κB activation with an increase in MDM2 ubiquitin ligase and the degradation of p21. In general, these findings further indicate that the NF-κB pathway is a key point of CRC processes involving EMT and the angiogenic process.

According to the report, Approximately 760 natural and synthetic molecular inhibitors of the NF-κB pathway have been developed (Vaiopoulos et al., 2013). A wide array of IKK inhibitors has been reported, but none have put into clinical practice. Until now, owing to the broad spectrum of the inhibitors,the nature of NF-κB inhibitors remains incompletely defined. However, first-in-class IKK-α-specific inhibitors are available. NF-κB activity is associated with chemoresistance and radioresistance, and curcumin was able to block this NF-κB activity (Sandur et al., 2009).

## Conclusion and Future Considerations

In addition to the above four signaling pathways, there are other important signaling pathways in the process of tumor development, such as p53 signaling pathway, Hippo signaling pathway, Hedgehog pathway, TGF-β pathway and JAK-STAT pathway. P53 signaling pathway is a typical pathway of abnormal disorder in tumors. Due to the complexity of p53 pathway, imperfect drug design methods and other restrictive conditions. Although some drugs targeting p53 have entered the clinical stage, including APG-115 and UBX-0101, no drugs are on the market. Hippo signaling pathway is closely related to tumor immunity. YAP in the Hippo pathway is highly expressed and functions in the generation of regulatory T cells and plays a certain role in anti-tumor immunosuppression. On the other hand, the Hippo pathway can regulate the immune checkpoint molecule PD-L1, thereby enhancing the body’s immunity (Ma et al., 2017). Inhibitors targeting the Hedgehog pathway Vismodegib and Sonidegib were approved by the US Food and Drug Administration (FDA) in 2012 and 2015, respectively, for the treatment of basal cell carcinoma (BCC) and associated blastoma (MB), but to date there is no evidence that inhibitors of the Hedgehog pathway can treat the five cancers described above. A variety of TGF-β inhibitors have been developed to kill tumor cells. Fresolimumab, an inhibitor of TGF-β, has entered phase I clinical trials in breast cancer. Galunisertib has also entered phase II clinical trials in HCC. The JAK-STAT pathway is also involved in the genesis, progression, metastasis and drug resistance of tumors, especially STAT3 and STAT5 can continuously activate the survival, proliferation and invasion of tumor cells, which are of great interest in cancer biology. Ruxolitinib and Cucurbitacin I (JSI-24) inhibitors of JAK and STAT have been reported to treat solid tumors.

In addition to miRNAs, noncoding RNAs, such as long non-coding RNAs (lncRNAs) and circular RNAs (circRNAs), have also attracted extensive attention due to their important regulatory roles in tumor development involving in Wnt/β-catenin, PI3K/AKT/mTOR, Notch and NF-KB pathways (Anastasiadou et al., 2018). LncRNAs are currently considered to regulate gene expression mainly at transcriptional and post transcriptional levels. At the transcriptional level, the most common mechanism of lncRNAs regulating gene transcription is the direct interaction between lncRNAs and transcriptional complexes or DNA elements. At the post transcriptional level, lncRNAs regulates gene expression through the regulation of mRNA stability, mRNA splicing and modification, mRNA translation, protein stability and subcellular localization. Besides, CircRNAs and lncRNAs act as miRNA sponges to participate in the regulation of gene splicing, transcription and gene expression. As competitive endogenous ribonucleic acids (ceRNAs), circRNAs and lncRNAs can compete for miRNA binding to its response elements (MRes), thus affecting the expression of miRNA. In the Wnt/β-catenin pathway, lnc01133 regulates APC expression by acting as a miR-106a-3p ceRNA to affect Wnt/β-catenin pathway (Yang et al., 2018a). The researchers found that lncRNA CCAL regulates the progression of CRC through inhibiting activator protein APC2α activate Wnt/β-catenin signaling pathway. In addition, lncRNA BCAR4 has been found to directly interact with and stabilize β-catenin protein, which promote the progress of CRC. Interaction between β-catenin and lncRNAs can also influence its cellular localization, CYTOR by favoring the nuclear localization of β-catenin to promote CRC metastasis (Schwarzmueller et al., 2020). Other malignant tumors are no exception, LncRNA CCAT2 and UCA1 can activate Wnt and promote the development, migration and invasion of BC. The up regulation of lnc00968 promotes the growth, migration and invasion of non-small cell lung cancer cells by activating Wnt/β-catenin signaling pathway (Hu et al., 2018). A large number of studies have found that lncRNAs play an important role in PI3K/AKT/mTOR pathway. Importantly, lncRNAs are involved in the occurrence, development, metastasis and drug resistance of solid tumors, lncRNA-SNHG7 can target miR-34a and regulate PI3K/AKT/mTOR pathway to promote the occurrence and development of CRC. LncRNA TM4SF1-AS1 promotes the migration and invasion of LC cells by activating PI3K/AKT/mTOR signaling pathway. LncRNA-HNF1A-AS1, as a ceRNA, can activate PI3K/AKT/mTOR signal pathway by competitively binding miR-30b-3p to promote GC metastasis. This indicates that TM4SF1-ASS1, lncRNA-SNHG7 and LncRNA-HNF1A-AS1 may be a new target for molecular therapy of tumors. LncRNA PICSAR appears to function as miR-588 sponge in HCC cells, activating PI3K/AKT/mTOR signaling pathway and plays a carcinogenic role. It can be used as a potential prognostic biomarker and therapeutic target of HCC. LncRNA HOTAIR enhances the drug resistance of GC cells by regulating PI3K/AKT signaling pathway (Liu et al., 2020). In the Notch signaling pathway, lncRNA FAM83H-AS1 and lncRNA FOXD2-AS1 can regulate the Notch signaling pathway and promote the development of CRC (Li et al., 2018b). In the NF-kB signaling pathway, lncRNA NKILA can interact with NF-kB, participating in the negative feedback regulation of NF-kB, and acts as a tumor suppressor gene in BC.

CircRNAs also plays an important role in the occurrence, migration and invasion of malignant tumors by affecting Wnt/β-catenin, PI3K/AKT/mTOR and Notch pathways. In HCC, circRNA DEND4C enhances the expression of TCF4 through activating Wnt/β-catenin pathway and regulates the malignant behavior of HCC cells. Overexpression of circ0067934 increases FZD5 expression by activating miR-1324, which leads to Wnt/β-catenin pathway is activated. There is evidence that circ_001946 up-regulates SIRT1 expression, SIRT1 exerts promotive effects on Wnt/β-catenin cascade by targeting miR-135a-5p promotes the proliferation of LC cells. Circ_0006427 up regulates the expression of DKK1 and Wnt/β-catenin signaling pathway is inactivated (Li et al., 2019). Overexpression of circ103809 accelerates the progression of BC by regulating PI3K/AKT/mTOR signaling pathway *in vivo* and *in vitro*. CircRNA APLP2 can activate Notch signaling pathway in CRC by targeting miR 101-3p to promote proliferation and metastasis (Arab et al., 2017). In view of the importances of lncRNAs and circRNAs in signaling pathways, it suggests that lncRNAs and circRNAs may be potential targets for cancer treatment and diagnostic indicators for predicting therapeutic response.

Currently, scientists have designed an increasing number of drugs targeting signaling molecules. In clinical practice, molecular targeted therapy does reduce the toxic side effects of drugs to greatly improve the quality of patient survival, and can substantially prolong the survival time of patients, which brings new hope to many patients with cancer (Alzahrani, 2019). But targeted drugs still have certain limitations. First, not all patients are suitable for the use of targeted agents, only those patients with appropriate genetic mutations will respond to the targeted drug. And, there are many rare mutations that have not yet been developed for drugs. In addition, there is a subset of patients no responding to the targeted drugs, even if they have the corresponding mutation. The most common in the clinical is the rapid development of resistance in tumor cells to targeted drugs (Alzahrani, 2019), and its mechanisms are various, for example, crosstalk between signaling pathways, reactivation of downstream signals, and activation of alternative pathways and so on. The establishment of compensatory cell signaling pathway is an important mechanism that is, after one signaling pathway inhibited by drug; another signaling pathway is possibly activated to stimulate cell proliferation. Therefore, several molecularly targeted drugs are often used in combination or with other therapeutic modalities in clinical practice to improve drug efficacy and circumvent drug resistance. For example, Rapamycin inhibitors of PI3K/AKT/mTOR signaling pathway and SMO inhibitors of hedgehog signaling pathway significantly delayed the development of drug resistance in Medulloblastoma (Krishnamurthy and kurzrock, 2018; Buonamici et al., 2010). It has also been shown that the combination of drugs targeting signaling pathways and drugs interfering with autophagy to treat tumors can also achieve better therapeutic outcomes. Preclinical studies have found that the combination of the autophagy inhibitor hydrochloroquine (HCQ) and the targeting AKT drug MK2206 can treat solid tumors. In addition, the combination of signaling pathway targeted drugs with immunotherapy drugs is also an effective way to circumvent drug resistance (Alzahrani, 2019).

In fact humans have an extremely limited knowledge of signaling pathways. At present, in the internet age, big data is considered to be a powerful tool to broaden and deepen people’s understanding of targeted drugs. We guess, if gene sequences of every cancer patient in the world and the therapeutic effects by drugs targeting one or more genes mutations are documented and shared, for patients with the same genetic mutation, the key genetic mutations will be more quickly identified and the best treatment to the patient will be formulated. In addition, by big data, scientists could further understand the molecular mechanisms in the genesis and development of tumors, which is very helpful for developing a new generation of targeted drugs. Thus, it seems a powerful measure for pushing molecular targeting research, which has shown great promise but has always been hard to see in practice. At that, as the cost of gene sequencing drops, more cancer patients are willing to have their genes sequenced, the explosion of genomic data makes this idea a reality.
